# The physiological effect of heavy metals and volatile fatty acids on *Methanococcus maripaludis* S2

**DOI:** 10.1186/s13068-018-1302-x

**Published:** 2018-11-02

**Authors:** Annalisa Abdel Azim, Simon K.-M. R. Rittmann, Debora Fino, Günther Bochmann

**Affiliations:** 1 Institute for Environmental Biotechnology, IFA Department Tulln, University of Natural Resources and Life Sciences, Vienna, Austria; 2 Archaea Physiology & Biotechnology Group, Archaea Biology and Ecogenomics Division, Department of Ecogenomics and Systems Biology, Universität Wien, Althanstraße 14, 1090 Vienna, Austria; 3 Department of Applied Science and Technology (DISAT), Politecnico di Torino, Turin, Italy; 4 Center for Sustainable Future Technologies, Istituto Italiano di Tecnologia, Turin, Italy

**Keywords:** Archaea, Methanogen, Metabolism, Closed batch, Copper, Zinc, Acetate, Propionate

## Abstract

**Background:**

Methanogenic archaea are of importance to the global C-cycle and to biological methane (CH_4_) production through anaerobic digestion and pure culture. Here, the individual and combined effects of copper (Cu), zinc (Zn), acetate, and propionate on the metabolism of the autotrophic, hydrogenotrophic methanogen *Methanococcus maripaludis* S2 were investigated. Cu, Zn, acetate, and propionate may interfere directly and indirectly with the acetyl-CoA synthesis and biological CH_4_ production. Thus, these compounds can compromise or improve the performance of *M. maripaludis*, an organism which can be applied as biocatalyst in the carbon dioxide (CO_2_)-based biological CH_4_ production (CO_2_-BMP) process or of methanogenic organisms applied in anaerobic digestion.

**Results:**

Here, we show that Cu concentration of 1.9 µmol L^−1^ reduced growth of *M. maripaludis*, whereas 4.4 and 6.3 µmol L^−1^ of Cu even further retarded biomass production. However, 1.0 mmol L^−1^ of Zn enhanced growth, but at Zn concentrations > 2.4 mmol L^−1^ no growth could be observed. When both, Cu and Zn, were supplemented to the medium, growth and CH_4_ production could even be observed at the highest tested concentration of Cu (6.3 µmol L^−1^). Hence, it seems that the addition of 1 mmol L^−1^ of Zn enhanced the ability of *M. maripaludis* to counteract the toxic effect of Cu. The physiological effect to rising concentrations of acetate (12.2, 60.9, 121.9 mmol L^−1^) and/or propionate (10.3, 52.0, 104.1 mmol L^−1^) was also investigated. When instead of acetate 10.3 mmol L^−1^ propionate was provided in the growth medium, *M. maripaludis* could grow without reduction of the specific growth rate (*µ*) or the specific CH_4_ productivity (qCH_4_). A combination of inorganic and/or organic compounds resulted in an increase of *µ* and qCH_4_ for Zn/Cu and Zn/acetate beyond the values that were observed if only the individual concentrations of Zn, Cu, acetate were used.

**Conclusions:**

Our study sheds light on the physiological effect of VFAs and heavy metals on *M. maripaludis*. Differently from *µ* and qCH_4_, MER was not influenced by the presence of these compounds. This indicated that each of these compounds directly interacted with the C-fixation machinery of *M. maripaludis*. Until now, the uptake of VFAs other than acetate was not considered to enhance growth and CH_4_ production of methanogens. The finding of propionate uptake by *M. maripaludis* is important for the interpretation of VFA cycling in anaerobic microenvironments. Due to the importance of methanogens in natural and artificial anaerobic environments, our results help to enhance the understanding the physiological and biotechnological importance with respect to anaerobic digestion, anaerobic wastewater treatment, and CO_2_-BMP. Finally, we propose a possible mechanism for acetate uptake into *M. maripaludis* supported by in silico analyses.

**Electronic supplementary material:**

The online version of this article (10.1186/s13068-018-1302-x) contains supplementary material, which is available to authorized users.

## Background

Methane (CH_4_) is one of the most important and most used energy gases worldwide [1]. CH_4_ is produced by strictly anaerobic methanogenic archaea (methanogens), which contribute to the global carbon cycle with approximately 1 Gt of CH_4_ produced per year [2]. The carbon and energy metabolism of methanogens is streamlined for the conversion of a restricted number of C1 and C2 substrates, i.e. carbon dioxide (CO_2_) and molecular hydrogen (H_2_), formate, acetate, methanol, methylamines, and methoxylated compounds. Among the methanogens, the physiology and metabolism of *Methanococcus maripaludis* S2 harbours intriguing characteristics, including a high specific growth rate (*µ*), mild growth temperature (35–39 °C), genetically manipulability due to the presence of selectable markers [3], and an effective method for transformation [4]. These features make *M. maripaludis* an excellent laboratory microorganism for physiological and biotechnological studies [5]. Being an autotrophic, hydrogenotrophic methanogen, *M. maripaludis* could be applied for CO_2_ mitigation through the CO_2_-based biological CH_4_ production (CO_2_-BMP) process [6–8]. Furthermore, *M. maripaludis* was suggested to be applicable for wastewater treatment, amino acid production, and value-added product synthesis [9]. Despite the enormous biotechnological potential of methanogenic archaea in general [6, 8, 10–13] and of *M. maripaludis* in particular [9, 14, 15], the physiological knowledge with respect to the putative toxicology towards heavy metals such as cadmium, chromium, copper (Cu), mercury, uranium, zinc (Zn), and volatile fatty acids (VFAs), e.g. acetate and propionate, is still limited.

The inhibitory effects of heavy metals and VFAs on biological methanogenesis have been investigated in mixed and pure cultures [16–19]. Many surveys on biogas production from anaerobic digestion examined the mutual influence of heavy metals and VFA degradation [20–23]. VFAs are known to possess antimicrobial properties that are responsible for alterations in membrane functions like membrane fusion induction, inhibition of amino acids transport, and uncoupling of chemiosmotic phosphorylation [24]. These alterations are due to the interference of organic acids with the establishment and maintenance of a functional pH gradient across the membrane, that can lead to inhibition of methanogenesis [25]. The impact of various concentrations of VFAs on methanogenic communities’ variation during anaerobic digestion was also largely investigated [25–27]. However, VFAs do not seem to modify the composition of the methanogenic population in anaerobic digesters [27].

With respect to pure cultures of methanogens, a tolerance towards VFAs > 60 mg L^−1^ was observed in *Methanosarcina barkeri*, *Methanothermobacter marburgensis*, and *Methanobacterium formicicum* [18]. Besides the toxic effects that these compounds may cause, acetate can be a source of carbon in the metabolism of autotrophic methanogens and enhance their biomass production, depending on the microbial species. In *M. marburgensis*, acetate can be assimilated and integrated in amino acids although it is not required for growth [28]. In *Methanothermococcus okinawensis*, acetate does not promote growth [29]. In *M. maripaludis*, acetate serves as a precursor for acetyl-CoA, which is synthetized via an AMP-forming acetate-CoA synthetase [30, 31] and is subsequently assimilated into cell carbon; however, aceticlastic methanogenesis does not occur [32, 33]. While methanogenesis and carbon fixation into biomass compete for CO_2_ during growth of *M. maripaludis* on H_2_/CO_2_, changes in acetate concentrations may alter the cellular carbon assimilation to the advantage of CH_4_ production. Moreover, it was shown that VFAs own an amplifying effect on the inhibitory activity of transition metals in pure cultures of various microorganisms such as *Escherichia coli*, *Bacillus subtilis*, and *Pseudomonas aeruginosa* [34]. Thus, VFAs act as transporters for transition metals through the permeable cytoplasmic membrane, inducing an increased entry of metals into the cell.

Zn and Cu are involved in the metabolism of many methanogens with a defined role in cell growth and methanogenesis [17]. There, Zn could be growth stimulating [17]. Indeed, RNA polymerase and other biosynthetic enzymes need Zn ions. Zn is the metal coordinator of a cysteine at the N-terminal of the subunit B of the heterodisulfide reductase (Hdr) complex in *M. marburgensis* [35]. Hdr provides one pair of high potential electrons to reduce the heterodisulfide of coenzyme B and coenzyme M (CoB-S-S-CoM) and one pair of low potential electrons, which are required for producing a reduced ferredoxin which is in turn fundamental for CO_2_ reduction in the first step of CO_2_ fixation in the methyl branch of the Wood–Ljungdahl pathway. Zn was also found in the active site of the methyl-tetrahydromethanopterin (H_4_MPT) CoM methyltransferase (Mtr) enzyme of *Methanosarcina barkeri* [36–39]. Moreover, Cu and Zn possibly inhibit the synthesis of acetyl-CoA via carbon monoxide dehydrogenase/acetyl-CoA synthase (CODH/ACS) by replacing the proximal nickel (Ni) site of the bimetallic Ni–Ni centre in the A cluster of the ACS enzyme [40–42]. The susceptibility of the Ni–Ni centre to Cu or Zn substitution depends on the conformational state of the α subunit of the ACS and increases with this order Cu > Zn [41].

**Fig. 1:**
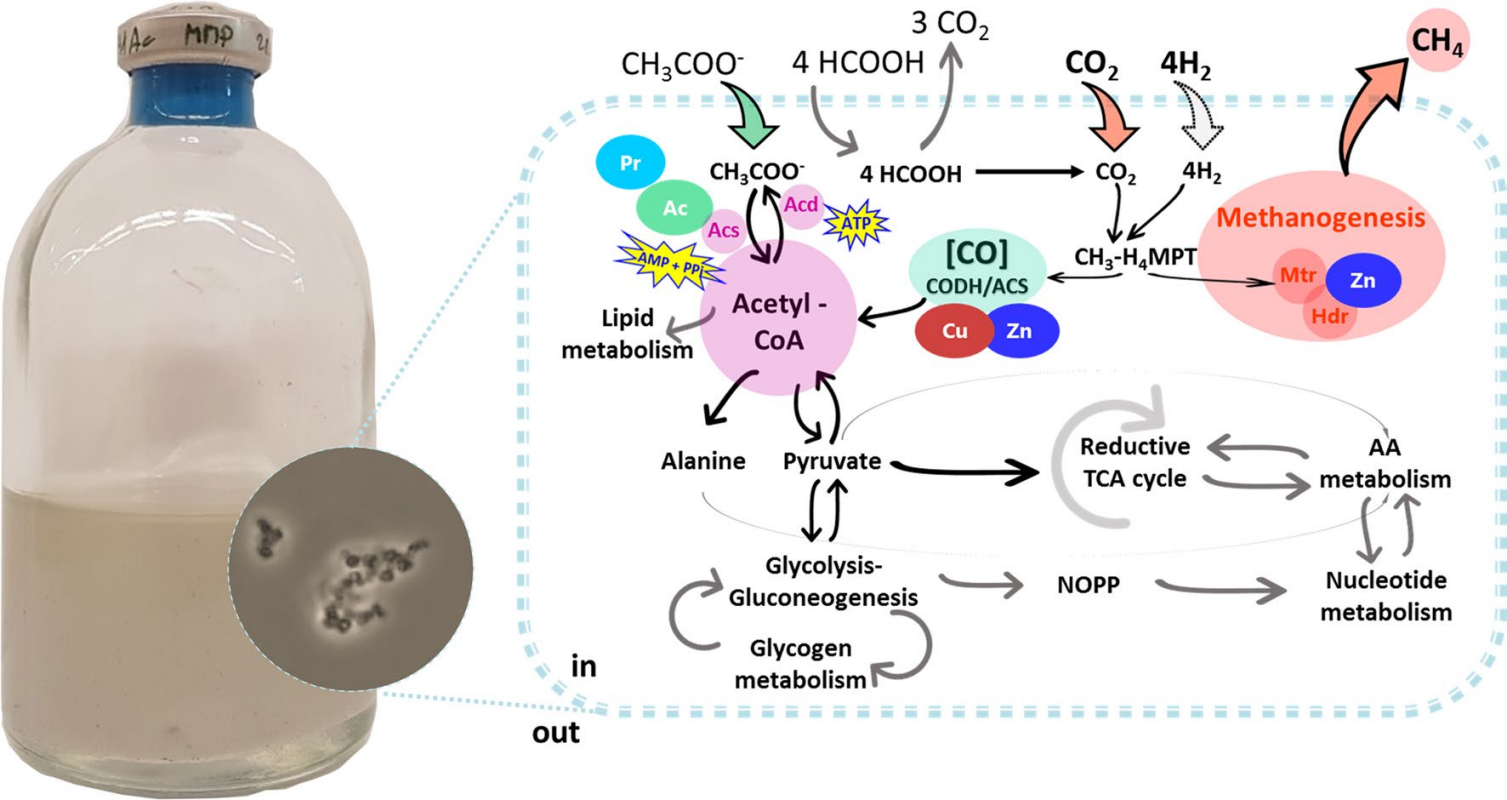
Schematic representation of the interactions in the metabolisms of *M. maripaludis* in closed batch cultures and the compounds tested in the present study. *Hdr* heterodisulfide reductase, *Mtr* methyl transferase, *Acs* acetyl-CoA (AMP-forming), *Acd* acetyl-CoA synthetase (ADP-forming), *ATP* adenosine triphosphate formation, *AMP + PPi* adenosine monophosphate and pyrophosphate from ATP hydrolysis, *AA* amino acid, *NOPP* non-oxidative pentose phosphate, *TCA* tricarboxylic acid, *CH*_*3*_*-HMPT* methyl-tetrahydromethanopterin. The present image was inspired by [9, 73]

The novelties of this work are the assessment of individual and combined effects of two heavy metals, Cu and Zn, and two VFAs, acetate and propionate, on growth and CH_4_ productivity of *M. maripaludis* S2 grown in closed batch cultivation mode on H_2_/CO_2_ (Fig. 1). *M. maripaludis* was tested at concentrations of these compounds higher then physiologically required. Moreover, to determine the role of acetate, we examined the effect of its deprivation. Although Cu, Zn, and acetate are known to be involved in pathways leading to acetyl-CoA formation and methanogenesis, the role of propionate had not yet been investigated. Hence, we investigated whether propionate could be used to stimulate growth and CH_4_ production of *M. maripaludis*. Moreover, the effects of acetate or/and propionate, in combination or without Cu or Zn, on the growth and CH_4_ productivity of *M. maripaludis* were examined in the light of the consolidated knowledge that was available in literature with respect to the role of acetate, propionate, Cu, and Zn during methanogenesis. The findings of our study have implications for improving biogas production from anaerobic digestion as well as for CH_4_ production during CO_2_-BMP and shed light on the ecophysiological importance of methanogens in VFA cycling in anaerobic environments.

## Results and discussion

*Methanococcus maripaludis* S2 was grown in defined m141 medium. The absence of vitamins, Cu, and Zn compounds did not affect growth and productivity (Additional file 1). Therefore, the m141 medium was subsequently used as the standard medium for all closed batch experiments.

### Growth of *M. maripaludis* is improved by addition of Zn

Zn and Cu, as well as the other heavy metals, originate from natural and anthropogenic sources, and are found in Earth’s crust. These compounds are spread in the environment through, e.g. volcanic activity, forest fires, atmospheric deposition, and human activities [43]. Domestic effluents and agricultural and industrial activities, such as mining, refining, combusting, lead to environmental contamination by metals [44]. Zn and Cu are considered trace elements together with other metals since their functions in many physiological and biochemical reactions are vital for the functioning of the metabolism. The biological availability depends on chemical, physical, and biological factors [45]. The effect of Zn and Cu toxicity on methanogenesis was widely studied in the context of anaerobic digestion of sewage sludge, but only few studies did yet cover the effect of those heavy metals on pure culture of microorganisms.

**Fig. 2:**
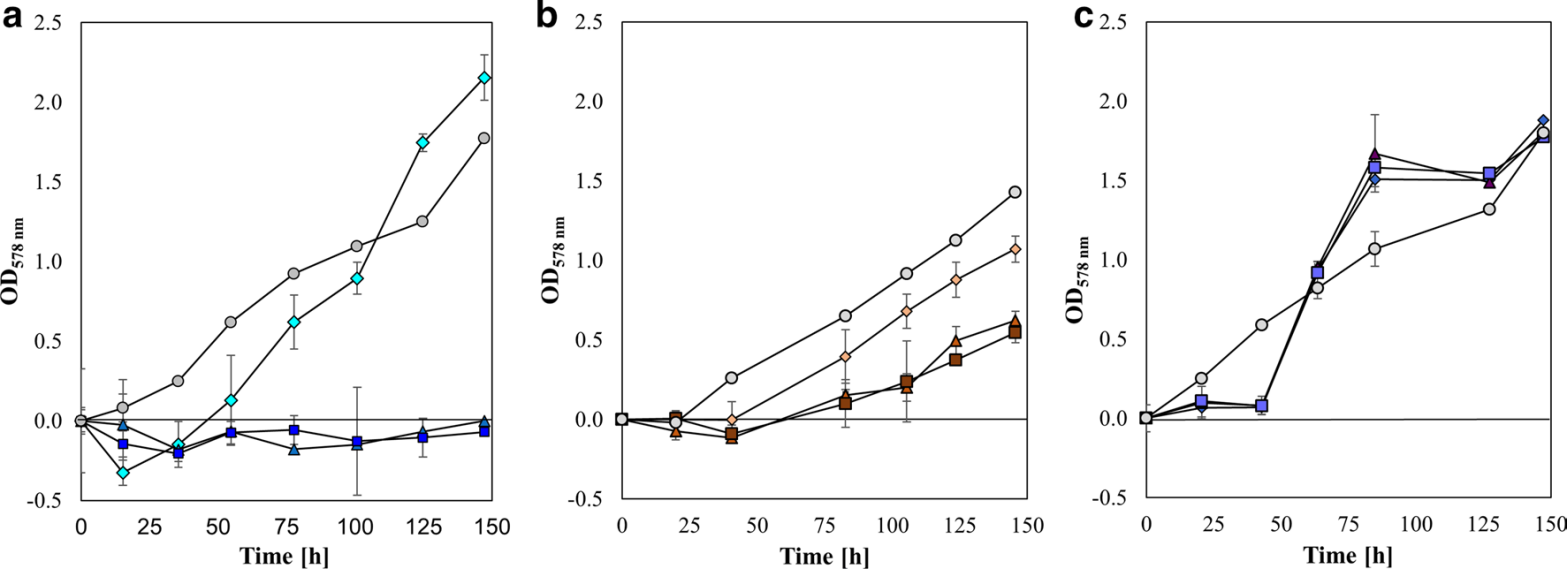
OD_578 nm_ curves of *M. maripaludis* 50 mL culture at 37 °C, 140 rpm, 2.9 bar with Zn, Cu, and Zn and Cu (*n* = 4). *M. maripaludis* was grown on m141 medium as positive control (  , *n* = 3), **a** on 1 mmol L^−1^ of Zn (  ), on 2.4 mmol L^−1^ of Zn (  ), on 3.9 mmol L^−1^ of Zn (  ); **b** on 1.9 µmol L^−1^ of Cu (  ), 4.4 µmol L^−1^ of Cu (  ), 6.3 µmol L^−1^ of Cu (  ), **c** on 1 mmol L^−1^ of Zn and 1.9 µmol L^−1^ of Cu (  ), on 1 mmol L^−1^ of Zn and 4.4 µmol L^−1^ of Cu (  ), on 1 mmol L^−1^ of Zn and 6.3 µmol L^−1^ of Cu (  )

The individual and combined effects of increasing Zn and Cu concentrations on the growth of *M. maripaludis* were determined by monitoring OD changes (Fig. 2). The results indicated that growth of *M. maripaludis* was completely inhibited at 2.4 and 3.5 mmol L^−1^ of Zn (Fig. 2a). An addition of 1.0 mmol L^−1^ of Zn did only result in a markedly longer lag phase compared to the positive control (medium m141). Conversely, the responsiveness of this microorganism to Cu toxicity seems to be higher since the Cu concentrations under which *M. maripaludis* growth was still observable were of two order magnitudes lower than for Zn. Despite the recommended concentration of Cu of 0.6 µmol L^−1^ in methanogenium medium (DSMZ medium 141), *M. maripaludis* could still grow at 1.9, 4.4, and 6.3 µmol L^−1^, though the maximum OD values were 1.07 ± 0.08, 0.62 ± 0.06, 0.54 ± 0.06, respectively, compared to 1.43 ± 0.001 in the positive control (medium m141) (Fig. 2b). Therefore, a direct correlation between increasing Cu concentration in the growth medium and a lower final biomass concentration of *M. maripaludis* seems to be very likely. These results are in agreement with earlier reports on *Moorella thermoacetica*, where a concentration of Cu (CuCl_2_) higher than 1 µmol L^−1^ in the medium is deleterious, and 10 µmol L^−1^ of Cu caused a 50% reduction of the CODH and ACS activity due to an alteration of the Cu content in the enzymes active site [46]. However, there are also species which showed a higher tolerance to Cu. The Cu concentrations needed for reducing methanogenesis by 50% in *M. formicicum*, *Methanobacterium hungatei*, *M. barkeri*, and *M. marburgensis* were 26, 30.5, 17, and 35.7 µmol L^−1^, respectively [18]. The elevated resistance of *Methanothermobacter thermoautotrophicum* KHT-2 to Cu was shown by a complete growth inhibition at 1 mmol L^−1^ of Cu [47]. One main issue in measuring OD in presence of elevated concentrations of Cu is related to the formation of agglomerates in the medium due to Cu-sulphide precipitation. The sulphide (Na_2_S·9H_2_O) concentration in the medium is 0.5 g L^−1^. However, to account for this effect the absorbance of the negative control (sample containing medium only) was subtracted from the replicates. The formation of Cu-sulphide precipitates fluctuated over time. This effect could mitigate the toxicity of Cu as in the Cu-sulphide form the compound is not available for the microorganisms [48, 49]. By combining Zn and Cu at each selected concentration (Fig. 2c), the lag phase lasted 40 h. The three growth curves overlap almost perfectly. Moreover, the maximum OD values range between 1.88 ± 0.09 and 1.78 ± 0.06. Therefore, the addition of 1.0 mmol L^−1^ of Zn obviated Cu toxicity on *M. maripaludis*. This effect could be explained by the fact that Cu and Zn are both divalent cations, which often compete for the same transporter in many cell types. It could be expected that the addition of Zn to the medium retards Cu entry into the cell. In our study, *M. maripaludis* grew at concentrations of Zn that were 45 and 100 times higher than the concentration applied in growth experiments using *M. marburgensis* or *M. barkeri*, respectively [39]. This indicates a strong requirement for this metal by *M. maripaludis*.

### Propionate stimulates growth and CH_4_ production of *M. maripaludis*

**Fig. 3:**
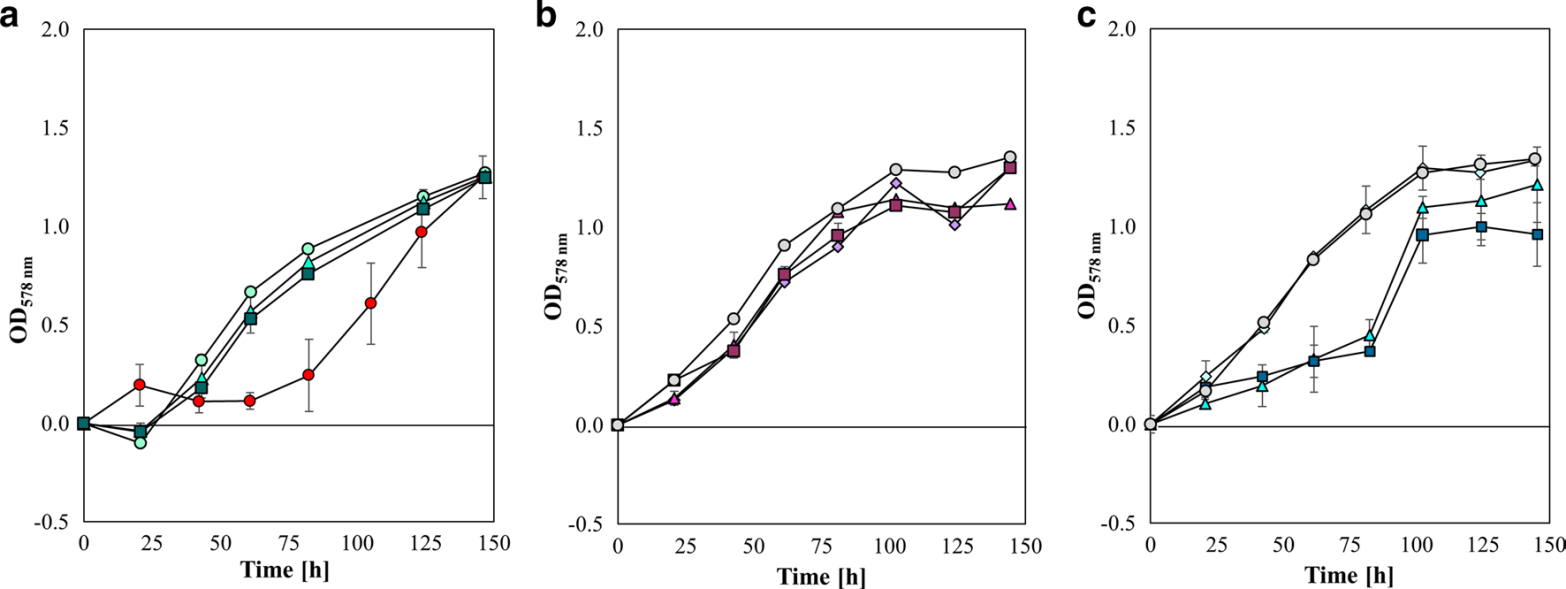
OD_578 nm_ of *M. maripaludis* 50 mL culture at 37 °C, 140 rpm, 2.9 bar with acetate and without acetate, with propionate and acetate, with propionate only (*n* = 4). *M. maripaludis* was grown on: **a** 12.2 mmol L^−1^ as positive control (  *n* = 3), 60.9 mmol L^−1^ of acetate (  ), 121.9 mmol L^−1^ of acetate (  ), without acetate (  ); **b** 12.2 mmol L^−1^ of acetate as positive control (  , *n* = 3), 12.2 mmol L^−1^ of acetate and 10.4 mmol L^−1^ of propionate (  ), 60.9 mmol L^−1^ of acetate and 52.0 mmol L^−1^ of propionate (  ), 121.9 mmol L^−1^ of acetate and 104.1 mmol L^−1^ of propionate (  ); **c** 12.2 mmol L^−1^ of acetate and 10.4 mmol L^−1^ of propionate as positive control (  , *n* = 3), 10.4 mmol L^−1^ of propionate (  ), 52.0 mmol L^−1^ of propionate (  ), 104.1 mmol L^−1^ of propionate (  )

Different concentrations of acetate were used to stimulate or inhibit growth (Fig. 3a). The possible presence of an aceticlastic pathway in *M. maripaludis* was excluded by omitting CO_2_ and carbonate from the growth medium (see Figure S2 in Additional file 1). The results show that acetate cannot be the only source of carbon for *M. maripaludis*. Additionally, we examined whether propionate could stimulate or inhibit *M. maripaludis* growth (Fig. 3b), or whether propionate could replace acetate (Fig. 3c), as a potential source of carbon. Growth curves show a great similarity among the different concentrations of acetate and the maximum OD corresponds to a value about 1.25 (Fig. 3a). Growth curves at different acetate concentrations are very similar to each other and reach the maximum OD of 1.25 (Fig. 3a). In the absence of acetate (0Ac), OD was found to reach similar final values but to substantially decrease through the time course, resulting in a longer lag phase. This observation is possibly due to the disruption of the correct metabolic operation, since acetate is an antimicrobial compound [50], and owns a key role as precursor of many cellular pathways supporting cellular homeostasis [51]. Although, *M. maripaludis* was still able to grow to the same final OD, the physiological potential of this microorganism was compromised due to acetate limitation. Intracellular acetate generation may be an explanation for *M. maripaludis* behaviour in the absence of acetate. *M. maripaludis* harbours genes for the ADP-forming acetyl-CoA synthetase (Acd; MMP0253) [52], also known as acetate-CoA ligase (ADP-forming), and the AMP-forming-type acetate-CoA synthetase (Acs; MMP0148) [51, 53]. The ADP-forming acetyl-CoA synthetase catalyses a reversible reaction, which is responsible for acetate formation and ATP synthesis from acetyl-CoA, ADP, and P_i_ in halophilic archaea [54], and in hyperthermophilic archaea, i.e. *Pyrococcus furiosus* [55]. Despite that, an experimental demonstration of biosynthesis of acetate by *M. maripaludis* is missing. The addition of propionate to m141 medium did not compromise the growth of *M. maripaludis* at any concentrations (Fig. 3b). If acetate was not present in the medium, the growth curve followed the profile of the curve at 12.2 mmol L^−1^. However, when 60.9 and 121.9 mmol L^−1^ of acetate are replaced with 52.0 and 104.1 mmol L^−1^ of propionate in the medium (Fig. 3c), *M. maripaludis* growth resulted in a retardation of biomass production over the first 81 h of incubation, thereafter exponential growth commenced. The shape of these growth curves resembled a sigmoidal curve which may indicated a putative growth inhibition of *M. maripaludis* due to the presence of propionate. This effect could be explained by an interfering of propionate with the proton (H^+^) gradient across the cell membrane. The diffusion of weak acids like propionate, as well as acetate, into the cell may cause a drop in intracellular pH due to exclusion of the anions and the retention of the H^+^ which leads to uncoupling [56–59]. The excess of H^+^ in the cytoplasm would have to be extruded out to preserve a functional electrochemical gradient. H^+^ extrusion process requires ATP hydrolysis, causing a diminution in ATP availability. Consequently, acetate and propionate might act as “uncouplers” of the electrochemical gradient that affects the cellular homeostasis. Nevertheless, only propionate generated this peculiar growth curve suggesting that *M. maripaludis* needed an adaptation period before growth on propionate started. That may be ascribable to the presence of a specific mechanism of transport for acetate which also owns affinity for other carboxylic acids, such as propionate.

### The effect of VFAs and heavy metals on growth and CH_4_ production kinetics of *M. maripaludis*

**Fig. 4:**
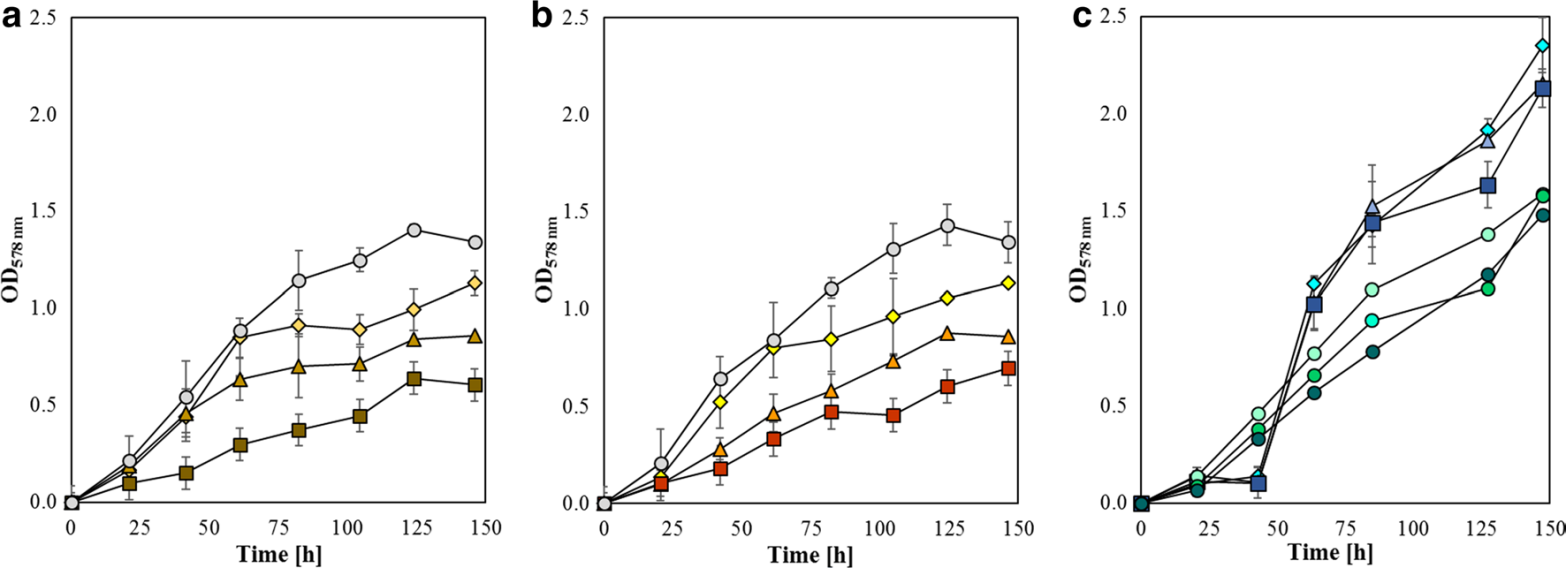
OD_578 nm_ curves of *M. maripaludis* 50 mL culture at 37 °C, 140 rpm, 2.9 bar with acetate combined with Cu and Zn, respectively (*n* = 4). *M. maripaludis* was grown on: **a** 60.9 mmol L^−1^ of acetate only as positive control (  , *n* = 3), 60.9 mmol L^−1^ of acetate with 1.9 µmol L^−1^ of Cu (  ), 4.4 µmol L^−1^ of Cu (  ) and 6.3 µmol L^−1^ of Cu (  ); **b** 121.9 mmol L^−1^ of acetate only as positive control (  , *n* = 3), 121.9 mmol L^−1^ of acetate with 1.9 µmol L^−1^ of Cu (  ), 4.4 µmol L^−1^ of Cu (  ) and 6.3 µmol L^−1^ of Cu (  ); **c** 12.2 mmol L^−1^ (  ), 60.9 mmol L^−1^ (  ), 121.9 mmol L^−1^ (  ) of acetate only as positive controls; on 12.2 mmol L^−1^ (  ), 60.9 mmol L^−1^ (  ), 121.9 mmol L^−1^ (  ) of acetate with 1 mmol L^−1^ of Zn

Given the distinct effect of Cu and Zn shown in Fig. 2, a series of closed batch experiments were performed to determine possible variations in the physiology of *M. maripaludis* with respect to acetate in combination with Cu or Zn, respectively. As discussed before, the presence of Zn and Cu can alter the catalytic rate of ACS/CODH complex, which is one of the two pathways used by *M. maripaludis* to synthetize acetyl-CoA. The other way is the AMP-forming acetyl-CoA synthetase (Acs) which is directly fuelled by acetate (Fig. 1). Although these two pathways are distinct, the combined supply of metals and acetate at higher concentrations may change the physiological response of *M. maripaludis*. Moreover, higher concentrations of VFAs may increase the uptake of metals into the cell. The growth curves resulting from these experiments are shown in Fig. 4. Despite the different acetate concentrations, the growth curves in Fig. 4a, b showed similar patterns. The reduction of biomass production compared to the positive controls (containing only 60.9 and 121.9 mmol L^−1^ of acetate) is directly dependent on increasing Cu concentration in the medium, but not on the concentration of acetate. This is also demonstrated by the similar values of the maximum OD which are 0.99 ± 0.11 (1Cu2Ac), 0.84 ± 0.13 (2Cu2Ac), 0.64 ± 0.06 (3Cu2Ac), 1.01 ± 0.20 (1Cu3Ac), 0.88 ± 0.03 (2Cu3Ac), 0.60 ± 0.04 (3Cu3Ac), respectively. Despite the presence of acetate, which supports growth via the ADP-forming acetyl-CoA synthetase, growth was compromised by the presence of rising Cu level which interferes with the ACS activity [46]. This may be an indication that the CO_2_-based acetyl-CoA production dominates over the acetate-based pathway even when a high concentration of acetate was available in the growth medium. Moreover, acetate can potentially form electron-neutral complexes with Cu as well as with Zn, which make both organic acids and metals more permeable to the cell membrane. This could cause a certain level of toxicity [34]. Concerning the use of 1.0 mmol L^−1^ of Zn with increasing concentration of acetate (Fig. 4c), the response of *M. maripaludis* seems to be homogenous and independent of the acetate content. The maximum OD values are higher than 2 (Fig. 2a). Supposing that ACS is effectively targeted by Cu in *M. maripaludis*, excess acetate, the precursor of acetyl-CoA (Fig. 1), did not counteract the inactivity of the ACS. It could it be that the CODH/ACS complex owns a prime role over the acetyl-CoA synthetase that is based on concomitant acetate and ATP consumption. However, the fact that acetate does not reduce Cu inhibition could also be seen as evidence against ACS as the primary target and it could be that other potential targets other than ACS are affected.

**Fig. 5:**
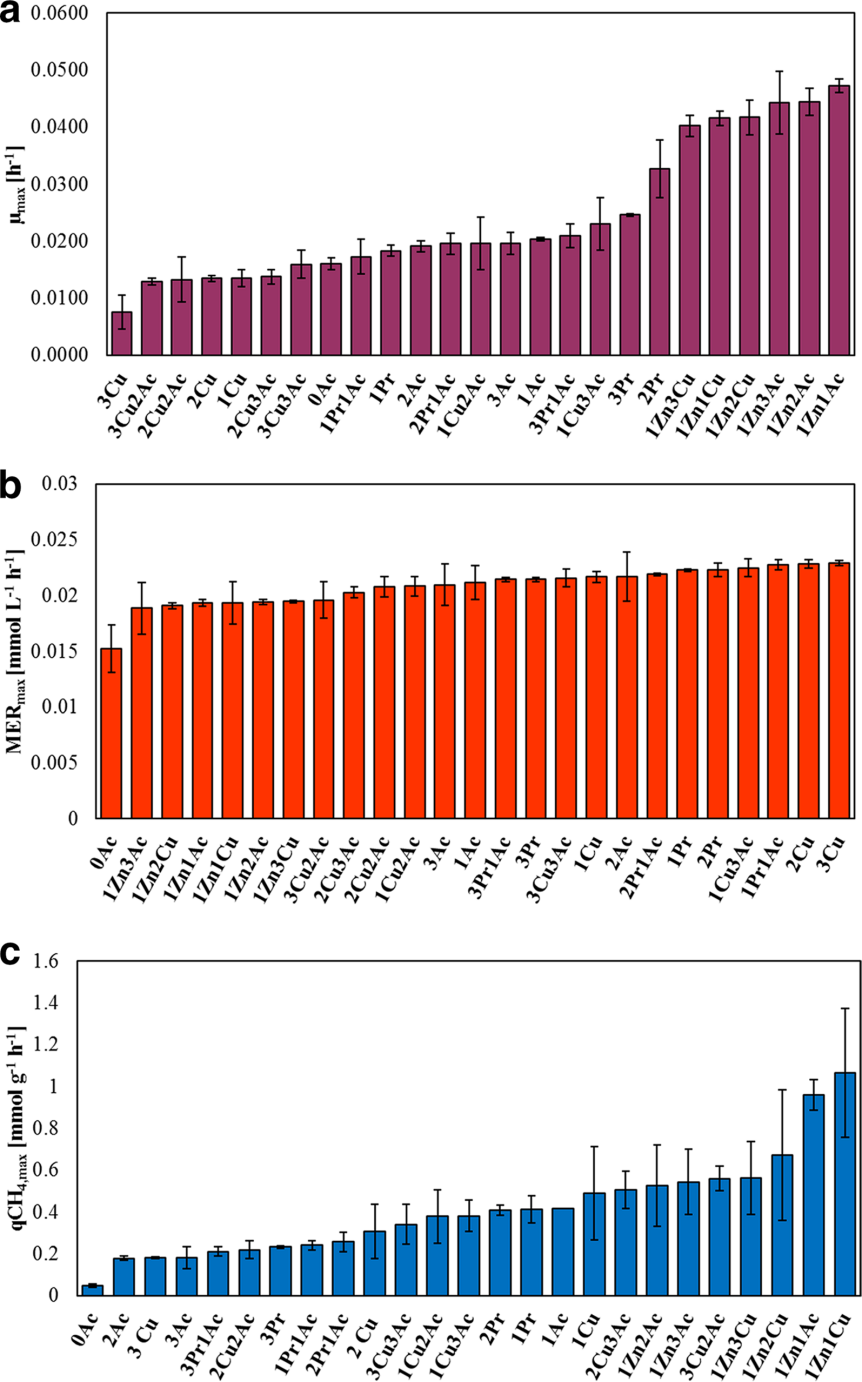
Histograms of **a** *µ*_max_, **b** MER_max_, and **c** qCH_4 max_ at each experimental condition applied in this study. *M. maripaludis* was grown at 37 °C, 140 rpm, 2.9 bar. Ac and Pr stand for acetate and propionate, respectively. The values 1, 2, 3 refer to the increasing concentrations of each compounds applied individually and simultaneously. (1,2,3) Zn: 1.0, 2.4, 3.9 mmol L^−1^; (1,2,3) Cu: 1.9, 4.4, 6.3 µmol L^−1^; (1,2,3) Ac: 12.2, 60.9, 121.9 mmol L^−1^, (1,2,3) Pr: 10.4, 52.0, 104.1 mmol L^−1^, 0Ac: without acetate

The kinetics of growth and CH_4_ production have been analysed and the results are presented in Fig. 5a, b. The impact of the different growth conditions that were experimentally addressed was less evident when MER_max_ was examined than when specific growth rate (*µ*_max_) was analysed (Fig. 5a). As a general consideration, these results all together indicate that Cu, Zn, acetate, and propionate have a major influence on the central metabolism of *M. maripaludis*, but neither the individual nor the combination of these elements seriously affected growth and biological CH_4_ production. More precisely, *µ*_max_ analysis highlighted a different physiological effect of the two metals in *M. maripaludis*. Indeed, the culture that grew at 6.3 µmol L^−1^ of Cu showed the slowest growth (0.0076 ± 0.0029), while that grown at 1.0 mmol L^−1^ of Zn and 12.2 or 60.9 or 121.9 mmol L^−1^ of acetate showed *µ*_max_ values more than double compared to the positive control (0.0204 ± 0.0003 h^−1^). The deleterious effect of Cu could be due to the formation of a Cu–Ni centre which produces an inactive form of ACS [40, 42, 60]. This inactivity is due to the poor nucleophilicity of Cu that prevents it from accepting the methyl group donated by the H_4_MPT from methanogenesis [9, 41]. Also, Zn can bind to the metal centre of ACS and thus interfere with its proper enzymatic function [42]. However, the results of growth (OD and *µ*_max_) indicated a different physiological effect of the two metals in *M. maripaludis*. Indeed, Zn did not inhibit growth of *M. maripaludis*, rather it enhanced it (Fig. 2a, 4c). The key to understand this difference may be that Cu does not only inhibit the ACS activity, but also that of CODH, preventing the CO oxidation reaction, whereas Zn supposedly interfered with the methyl transfer [41]. Moreover, Zn can replace Ni in the ACS active site when the protein conformation is open, e.g. when the enzyme is already involved in catalysis [60–62]. One may speculate that the excess of Zn is physiologically balanced through the involvement in many enzymatic catalyses and affinity as metal cofactor such as the Hdr, Mtr, and RNA polymerase [28–32, 34, 35], which favour the growth of *M. maripaludis*, rather than negatively affecting it. The presence of higher concentration of acetate (2Ac and 3Ac) did not alter *µ*_max_ compared to the positive control (1Ac) as expected on the base of the growth curves (Fig. 3a). The addition of propionate did not affect the *µ*_max_ too, except for the cultures with 52.0 and 104.1 mmol L^−1^ (2Pr and 3Pr). Here, a sudden biomass accumulation after the 81 h was visible (Fig. 3c).

With respect to CH_4_ productivity, the highest values were observed for the positive control having a MER_max_ value of 0.021 ± 0.002 mmol L^−1^ h^−1^. The lowest productivity (0.015 ± 0.002 mmol L^−1^ h^−1^) corresponds to the medium deprived of acetate (0Ac) while the highest values (0.023 mmol L^−1^ h^−1^) are represented by the cultures which were grown with 4.4 and 6.3 µmol L^−1^ of Cu (2Cu and 3Cu). Therefore, under acetate deprivation, *M. maripaludis* can still proliferate and reach the maximum OD, but the kinetics of CH_4_ production was compromised. Indeed, in the absence of acetate, the most easily accessible substrate for cell growth via acetyl-CoA production is CO_2_, but it needs to serve the combined carbon and energy metabolism of *M. maripaludis*. It may be possible that because of the competition between biomass generation and CH_4_ production, *µ*_max_ and MER_max_ became retarded. It is notable that all the cultures which have grown with Zn-amended medium possess a MER_max_ of 0.019 mmol L^−1^ h^−1^, which corresponds to a productivity reduction of 8–11% compared to the positive control (medium m141), in contrast to the elevated values of *µ*_max_ (Fig. 5a). This finding confirmed the crucial role of Zn in supporting cell growth on one side and indicates a probable inhibitory effect on methanogenesis on the other side. A possible explanation may be found in the interference of Zn abundance with more than one enzyme involved in the methanogenesis, e.g. the Hdr and Mtr. Indeed, the intermediate of methanogenesis, the methyl-H_4_MPT, which is transferred by Mtr to the CoM, is the source of the methyl group of acetyl-CoA during CO_2_ fixation. Therefore, the abundance of Zn may alter the equilibrium concentration in the mechanism that regulates the destiny of methyl-H_4_MPT in one direction rather than the other. The effect of Cu (1.9, 4.4, and 6.3 µmol L^−1^) on MER_max_ is almost homogenous at each concentration. However, when Cu was combined with acetate, the productivity decreased compared to the positive control (medium m141). Although the reason this occurs is not clear yet, a possible explanation for this may be the formation of Cu-acetate aggregates which make Cu more easily available for the microorganism [34]. Thus, also the effect of Cu would be intensified when driven by acetate through the cell membrane. Figure 5C shows that qCH_4,max_ values are due to the fluctuation of biomass concentration under different experimental conditions, since MER_max_ values are rather constants. Moreover, qCH_4,max_ profile is similar to the *µ*_max_ profile (Fig. 5a). The 0Ac sample owns the lowest value of qCH_4,max_ (0.047 ± 0.009 mmol g^−1^ h^−1^), which confirms that the catalytic power of *M. maripaludis* is heavily influenced when deprived of acetate. On the contrary, qCH_4,max_ was maximized by the presence of 1 mmol L^−1^ of Zn was in the medium (1Zn1Ac and 1Zn1Cu), achieving values of 0.96 ± 0.07 and 1.07 ± 0.31 mmol g^−1^ h^−1^. Thus, the beneficial effect of Zn on *M. maripaludis* growth (Fig. 2a) improved also its qCH_4_. Increasing the concentrations of Cu (1Cu 1.9, 2Cu 4.4, and 3Cu 6.3 µmol L^−1^) led to decreasing qCH_4,max_ values (0.49 ± 0.22; 0.31 ± 0.13; 0.181 ± 0.004 mmol g^−1^ h^−1^, respectively, Fig. 5c). The high susceptibility of *M. maripaludis* to this metal is proved again by these results. The effect of propionate on qCH_4,max_ is strictly connected to the concentration in the sample. qCH_4,max_ assumed similar values among the different concentration of acetate, indicating that this parameter is not dependent on the acetate concentration.

### Analysis of acetate and propionate uptake by *M. maripaludis*

**Fig. 6:**
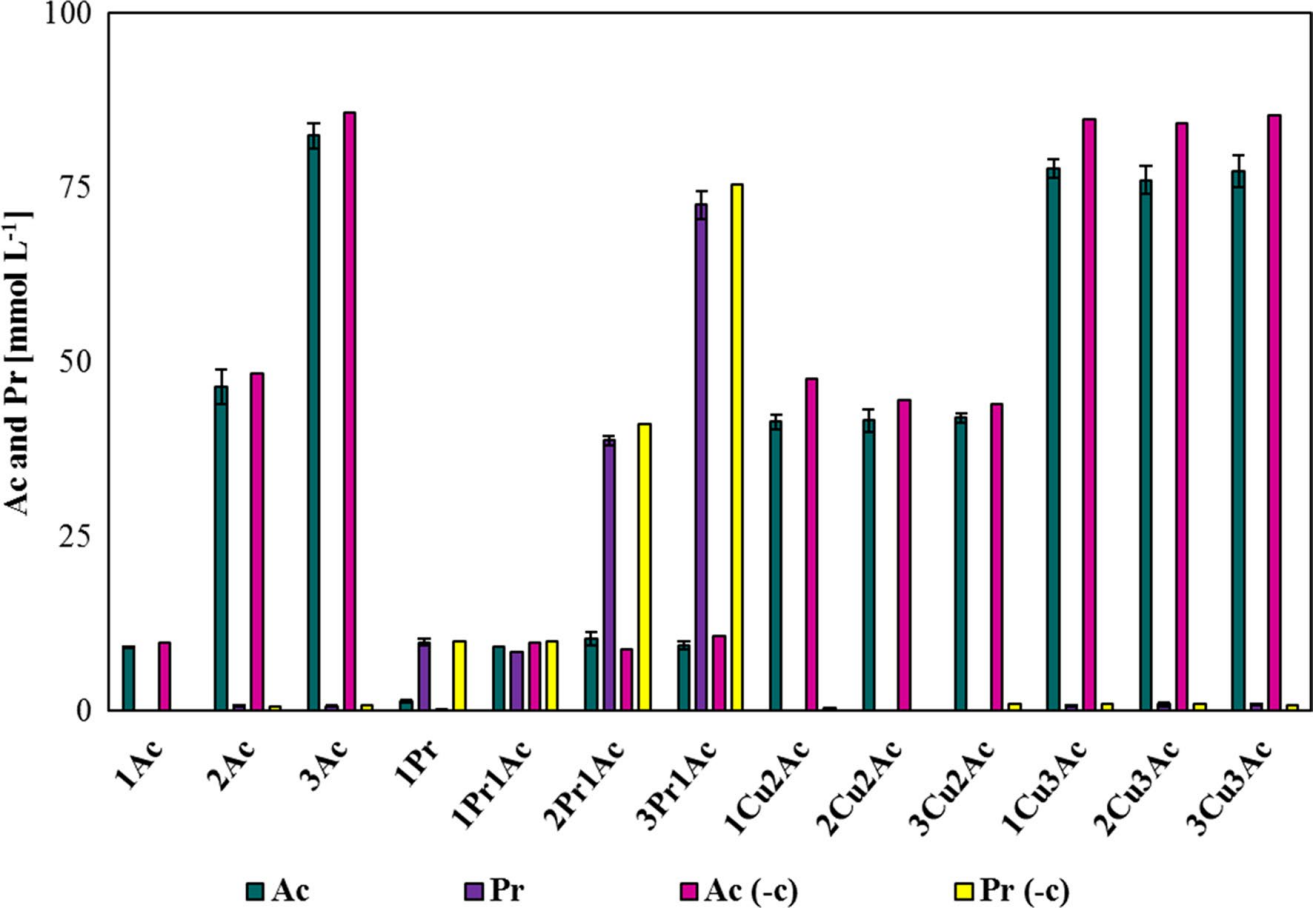
Concentration of acetate (Ac) and propionate (Pr) measured at the end of the closed batch cultivation of *M. maripaludis* 37 °C, 140 rpm, 2.9 bar. The values 1, 2, 3 refer to the increasing concentrations of each compounds applied individually and simultaneously. (1,2,3) Ac: 12.2, 60.9, 121.9 mmol L^−1^; (1,2,3) Pr: 10.4, 52.0, 104.1 mmol L^−1^ 12.2, 60.9, 121.9 mmol L^−1^; Cu (1.9, 4.4, 6.3 µmol L^−1^)

The values reported in Table 1 prove that both acetate and propionate are consumed among the different samples, with some exceptions represented by 1Pr. The HPLC analysis of acetate and propionate levels was performed at the end of the closed batch experiments. Although in 1Pr, acetate is supposed to be zero, the analysis revealed a concentration equal to 1.39 ± 0.27 mmol acetate L^−1^ in respect of 0.34 mmol acetate L^−1^ in the negative control (sample containing medium only). These data may support the hypothesis of endogenous acetate synthesis by the ADP-forming acetyl-CoA synthetase of *M. maripaludis* [52]. However, also 2Pr1Ac shows a value of acetate that is higher than the acetate content in the negative control. This is the only exception among the experiments where propionate was combined with acetate. Therefore, this deviation may be explained by a lower injection of acetate in the negative control sample during culture preparation. On the basis of these data, acetate synthesis would seem to be stimulated by the absence of acetate (1Pr) and it may explain the growth curve in Fig. 3c, which is almost the same of the curve corresponding to 12.2 mmol L^−1^ of acetate. Unfortunately, HPLC analyses of 2Pr and 3Pr, which show a different behaviour of *M. maripaludis* growth (Fig. 3c), were not available. Hence, we do not know if endogenous acetate production occurred at higher concentration of propionate, or if propionate may interfere with it given the longer lag phase (Fig. 3c). The graphical representation of Table 1 is illustrated in Fig. 6. By increasing acetate concentration in the medium from 12.2 to 121.9 mmol L^−1^, the absorbed concentration raised from 0.62 ± 0.04 to 2.41 ± 0.43 mmol L^−1^ respect to the negative control (Fig. 6). Despite these results, growth curves, *µ*, and MER are very similar among the different concentrations of acetate (Figs. 3a, 5a, b). The undissociated form of VFAs should be rather low compared to the anionic form at pH ≥ 8. Therefore, an increased acetate uptake may be regulated by a specific transport reaction. However, the amount of acetate that was assimilated is quite low and not even close to the 60% of extracellular acetate which would be possible to be assimilated [32]. Concerning propionate, the results indicate that *M. maripaludis* can recover this organic acid (1Pr1Ac, 2Pr1Ac, 3Pr1Ac), although the consumed concentrations are quite low compared to acetate, and the high standard deviations indicate a great variability among the replicates of this experiment. Moreover, as already mentioned above, the negative bar in 1Pr may be an indication of a possible acetate extrusion by *M. maripaludis* cells. Cu concentrations in 2Cu2Ac and 3Cu2Ac led to a reduction of acetate assimilation (Fig. 6). Interestingly, the same concentrations of Cu did not affect the recovery of acetate in 2Cu3Ac and 3Cu3Ac samples, which have similar values of acetate consumption. The data on acetate consumption in the presence of Cu are discordant among the different measurements. A possible explanation may be the formation of metal–acid aggregates that can enter the cell, which would improve at higher concentration of Cu and acetate or the complexation of elements and organic acids with sulphide. However, this marked difference is not consistent with the results of OD, *µ*_max_, and MER_max_ that were rather similar at 60.9 and 121.9 mmol L^−1^.

**Table 1:** Analysis of acetate (Ac) and propionate (Pr) concentrations at the end of each experiment

Sample	−c (mmol_Ac_ L^−1^)	Ac (mmol_Ac_ L^−1^)	−c (mmol_Pr_ L^−1^)	Pr (mmol_Pr_ L^−1^)
1Ac	9.81	9.19 ± 0.04	na	na
2Ac	48.34	46.47 ± 2.48	na	na
3Ac	85.74	82.43 ± 1.84	na	na
1Pr0Ac	0.34	1.39 ± 0.27	9.96	9.82 ± 0.49
1Pr1Ac	9.81	9.18	9.96	8.43
2Pr1Ac	8.92	10.39 ± 1.00	41.04	38.71 ± 0.67
3Pr1Ac	10.69	9.43 ± 0.57	75.48	72.51 ± 2.02
1Cu2Ac	47.63	41.40 ± 1.02	na	na
2Cu2Ac	44.47	41.60 ± 1.58	na	na
3Cu2Ac	43.85	41.98 ± 0.65	na	na
1Cu3Ac	84.77	77.72 ± 1.27	na	na
2Cu3Ac	84.16	76.08 ± 1.94	na	na
3Cu3Ac	85.37	77.38 ± 2.29	na	na

Batch cultivations of *M. maripaludis* were performed at 37 °C, 140 rpm, 2.9 bar. The values 1, 2, 3 refer to the increasing concentrations of each compounds applied individually and simultaneously. (1,2,3) Ac: 12.2, 60.9, 121.9 mmol L^−1^; (1,2,3) Pr: 10.4, 52.0, 104.1 mmol L^−1^ 12.2, 60.9, 121.9 mmol L^−1^; Cu (1.9, 4.4, 6.3 µmol L^−1^). The negative control (−c) refers to medium that did not receive inoculum of cells and therefore is used as a reference value

*na* not applied

### Mechanism of acetate transport into *M. maripaludis*

**Fig. 7:**
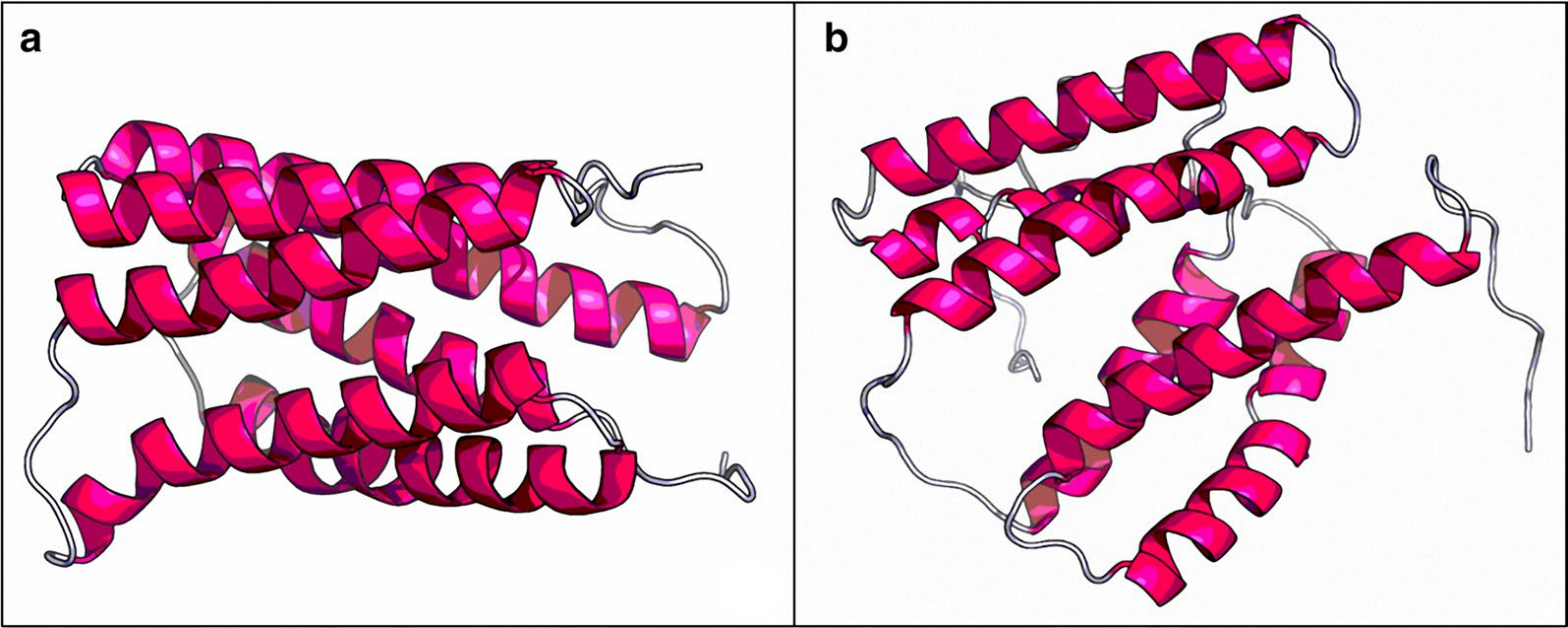
3-D model structure of **a** SatP and **b** MMP0348 based on their amino acid sequence (from RaptorX server)

It is of general interest to discuss the possible mode of action of acetate transport into the cytoplasm. Acetate is a strong protonophore which can readily diffuse into the cytoplasm even in dissociated form, if the pH is ≥ 7 [58, 59]. Evidence of acetate transport proteins has been shown in *Saccharomyces cerevisiae* [63] and *E. coli* [64], where the presence of these enzymes enhanced the acetate uptake. In *S. cerevisiae*, the Ady2p transmembrane protein was identified as responsible for acetate uptake when the cell was subjected to a shift from glucose to acetate. Acetate transport in *E. coli* is based on the Yahh protein. Yahh acts as an acetate-proton symporter, which is dependent on the transmembrane electrochemical potential. Moreover, Yahh is also able to recognize and uptake succinic acid though with a low affinity. Both Ady2p and Yahh belong to the GPR1/FUN34/SatP acetate transporter family. The knowledge about acetate transport into archaeal microorganism is poor. A pioneering study on this topic was performed with *Methanosarcina mazei* [65]. In this context, the role of *mm_0903* (Q8PYF9) gene in aceticlastic methanogenesis was shown and the relevance of this protein in settling the acetate threshold level in *Methanosarcina* spp. was hypothesized. Homologues to the MM_0903 protein were found also in non-aceticlastic methanogens, usually with an identity lower than 67% [65]. Although the present study does not specifically focus on acetate assimilation, we sought to identify a possible mechanism of transport in *M. maripaludis* which can explain the differences in acetate uptake, as evidenced in Fig. 6. The Blast search for MM_0903 resulted in 250 alignments, where 71 protein sequences corresponded to the archaeal domain. Among them, the GPR1/FUN34/YaaH family protein belonging to *Methanococcus vannielii* (*Mevan_1357*, A6URY2), matched MM_0903 with 60.8% of identity and an *E*-value of 3.8e−74. Therefore, by restricting the search for alignments to this methanococci related class, we were able to identify a matching protein sequence (identity of 80.3%, *E*-value of 1.48e−101) corresponding to *mmp0348* gene (Q6M0C3). MMP0348 corresponds to an uncharacterized protein of 197 amino acids belonging to *M. maripaludis.* Putative conserved domain on this sequence corresponds to the acetate transporter GPR1/FUN34/SatP evolutionary family belonging to *Candida lipolytica*, *S. cerevisiae, E. coli*, and *Aspergillus nidulans*. MMP0348 protein shows a very high domain-specific alignment (*E*-value of 3.71e−72) with SatP protein. SatP is associated to the succinate–acetate transport function in *E. coli* O157:H7 and MMP0348 revealed a 59% of identity of the sequences (see Additional file 2: Figure S3). Protein structural models of SatP and the hypothetical acetate transport protein MMP0348 of *M. maripaludis* S2 are illustrated in Fig. 7 [66–70]. The highest quality model for SatP protein with a *P-*value of 8.4e−06 is based on a succinate–acetate permease from *Citrobacter koseri* (see Additional file 2: Table S1) [71]. The best MMP0348 structure model was designed on a membrane protein for ammonium sensing (Amt protein template 6eu6A), which was also found among the selected templates for building SatP model (See Additional file 2: Table S1).

As for GPR1/FUN34/SatP family [63], also MMP0348 protein contains six transmembrane regions (Fig. 7) that may be involved in acetate transport. Moreover, MMP0348 protein is supposed to interact with MMP1274 (AMP-dependent synthetase and ligase) and MMP0148 or acsA (AMP-forming ACS) [72], but MM0348 was not yet biochemically characterized. The nature of these interactions is not clear since the presence of a unique gene cluster it has not been shown, but it is only a possibility based on homologous genes. However, if confirmed, the hypothetical acetate transporter (MMP0348) may act also as a precursor of acetyl-coA synthesis based on acetate. Supposing that, MMP0348 in *M. maripaludis* works as YaaH in *E. coli* [64], each molecule of acetate is co-translocated with H^+^. The result of this mechanism is the same as when acetic acid diffuses across the membrane and dissociates into acetate and the H^+^. The YaaH transporter can transport a broad range of substrates, e.g. borate, oxalate, pyruvate, lactate, malate, citrate, succinate, and acetate. However, formate, propionate, benzoate, salicylate, and butyrate were shown to possess an inhibitory effect (> 80%) on acetate uptake [64]. This suggested that all salts of the monocarboxylic acids acted as non-competitive inhibitors of acetate uptake in *E. coli*. Given the homology between YaaH and MMP0348, it is probable that there is a similar mechanism in *M. maripaludis.* Thus, it is possible to speculate about the reversible inhibition of propionate on cell growth because of the binding to MMP0348. This may explain the growth curves shape of 2Pr and 3Pr in Fig. 3c. As a non-competitive inhibitor, propionate could bind to the MMP0348 and enter the cell via the specific transport system. Furthermore, the analysis of *mm0348* gene expression and also the biochemical characterization of this putative transporter are nevertheless required to shed light on the possible interactions of MM0348 with the acetyl-CoA synthetase, and to prove its role in acetate and propionate uptake in *M. maripaludis*.

## Conclusions

The physiological effect of Cu and Zn, acetate, and propionate on *M. maripaludis* was examined. Differently from *µ* and qCH_4_, MER was not influenced by the presence of these compounds. This indicated that each of these compounds directly interacted with the C-fixation machinery of *M. maripaludis*. Interestingly, high Zn levels (1 mmol L^−1^) enhanced growth of *M. maripaludis* and mitigated the toxicity of Cu. Furthermore, as an alternative to acetate, propionate can be assimilated by *M. maripaludis*. Until now, the uptake of VFAs other than acetate, was not considered to be able to enhance growth and CH_4_ production of methanogens. The finding of propionate uptake by *M. maripaludis* is also important for the interpretation of VFA cycling in anaerobic microenvironments. Moreover, we propose a specific acetate transporter of *M. maripaludis*. However, the acetate transporter, which could possibly also be considered to act as propionate transporter, was only identified by using in silico analyses of the unclassified MM0348 protein. Our study attempted to shed light on the physiological effect of VFAs and heavy metals on *M. maripaludis*, but due to the importance of methanogens in natural and artificial anaerobic environments, our findings have implications for understanding the ecophysiology and CH_4_ production characteristics of these intriguing organisms.

## Methods

### Gases and chemicals

The gases, H_2_/CO_2_ (20 Vol.% CO_2_ in H_2_) and molecular nitrogen (N_2_), were from Air Liquide (Air Liquide GmbH, Schwechat, Austria). All other chemicals were of highest grade available.

### Culture preparation and maintenance

The mesophilic, autotrophic, hydrogenotrophic, and methanogenic organism *Methanococcus maripaludis* S2 [53] was used in all experiments. The methanogenium medium 141 DSMZ was modified as follows: 0.014 mg L^−1^ of FeSO_4_·7H_2_O instead of 2 mL Fe(NH_4_)_2_(SO_4_)_2_·6H_2_O, trypticase peptone, resazurin, and vitamins solutions were omitted from the medium. Trace element (TE) solution 141 DSMZ was prepared with the following modifications: instead of 0.500 g L^−1^ MnSO_4_·H_2_O, 0.585 g L^−1^ of MnCl_2_·4H_2_O were used, and both, CuSO_4_ and ZnSO_4_·7H_2_O were omitted from the solution. The modified medium (named as m141) was prepared and autoclaved in 1-L bottle and closed with a screw cap comprising three ports (Duran^®^, GL 45 blue PP screw cap with 3× GL 14 ports). A stock solution of 0.11 mol L^−1^ Na_2_S·9H_2_O was prepared and autoclaved in the aforementioned system. 2.38 mol L^−1^ NaHCO_3_ and 0.059 mol L^−1^ l-cysteine-HCl·H_2_O were prepared separately in 120 mL serum bottles (La-Pha-Pack, Langerwehe, Germany) as stock solutions, sealed, and deoxygenated by flushing them with N_2_ in a pressure range of 0.8–1.0 bar. All solutions were sterilized through autoclaving for 20 min at 121 °C.

### Closed batch cultures preparation

The autoclaved media were amended with 10 mL of TE solution and then purged through a filter with N_2_ to make it anoxic for 40 min. 50 mL of NaHCO_3_ stock solution, 5 mL of l-cysteine-HCl·H_2_O stock solution, and 18.75 mL Na_2_S·9H_2_O stock solution were then added by using stile syringes. The medium was then dispensed in 120 mL serum bottles (La-Pha-Pack, Langerwehe, Germany) that were previously flushed with N_2_ to remove residual oxygen, and then inoculated using 1 v/v of fresh culture, for a final volume of 50 mL per flask. All experiments were carried out in quadruplicates with one negative control (sample containing medium only) and one positive control (reference sample). Above-described operations were all performed inside a laminar flow (LaminAir^®^ HB 2472, Heraeus Instruments, Hallbergmoos, Germany). Immediately after inoculation, the bottles were pressurized with a H_2_/CO_2_ gas mixture between 2.7 and 3.1 bar as previously described [73]. Sterile syringe filters (w/0.2 cm cellulose, 514-0061, VWR International, USA), disposable hypodermic needles (Gr 14, 0.80 × 40 mm, 21 G × 1 1/2″, 80 × 25 mm, 21 G × 1″, Henke-Sass Wolf GmbH, Tuttlingen, Germany), and stopcocks (4-way with luer lock connections and male lock, Cole-Parmer, Illinois, United States) were used for feeding the cultures. After gassing, the flasks were incubated at 37 °C and 140 rpm (Multitron, Infors HT, Basel, Switzerland and Innova^®^ 44, Eppendorf AG, Hamburg, Germany). The headspace pressure in the serum bottles was measured daily using a digital manometer (2086P, 1–10 bar, Digitron, Italy). Residual CH_4_ was replaced every day by releasing the offgas and gassing the serum bottle headspace with H_2_/CO_2_ at a pressure between 2.7 and 3.1 bar. A volume between 0.9 and 1.1 mL of liquid sample was collected by using sterile syringes at regular intervals for monitoring biomass growth by measuring the optical density at 578 nm (OD_578_) using a spectrophotometer (DR 2800™ Portable Spectrophotometer, Hach, Australia). Every sampling operation was done inside the laminar. A summary of experiments performed within the frame of this study is shown in Table 2.

**Table 2:** Type of compounds used as potential inhibitors and its corresponding concentrations

Inhibitor type	#	Sample name	Compound and concentration
Inorganic^a^	1	1Zn	Zn 1.0 mmol L^−1^
	2	2Zn	Zn 2.4 mmol L^−1^
	3	3Zn	Zn 3.9 mmol L^−1^
	4	1Cu	Cu 1.9 µmol L^−1^
	5	2Cu	Cu 4.4 µmol L^−1^
	6	3Cu	Cu 6.3 µmol L^−1^
Organic and organic/organic	7	1Ac	Ac 12.2 mmol L^−1^
	8	2Ac	Ac 60.9 mmol L^−1^
	9	3Ac	Ac 121.9 mmol L^−1^
	10	1Pr	Pr 10.4 mmol L^−1^
	11	2Pr	Pr 52.0 mmol L^−1^
	12	3Pr	Pr 104.1 mmol L^−1^
	13	1Pr1Ac	(Pr 10.4 + Ac 12.2) mmol L^−1^
	14	2Pr1Ac	(Pr 52.0 + Ac 12.2) mmol L^−1^
	15	3Pr1Ac	(Pr 104.1 + Ac 12.2) mmol L^−1^
Combined inorganic/inorganic or combined inorganic/organic	16	1Zn1Cu	Zn 1.0 mmol L^−1^ + Cu 1.9 µmol L^−1^
	17	1Zn2Cu	Zn 1.0 mmol L^−1^ + Cu 4.4 µmol L^−1^
	18	1Zn3Cu	Zn 1.0 mmol L^−1^ + Cu 6.3 µmol L^−1^
	19	1Cu2Ac	Cu 4.4 µmol L^−1^ + Ac 60.9 mmol L^−1^
	20	2Cu2Ac	Cu 4.4 µmol L^−1^ + Ac 60.9 mmol L^−1^
	21	3Cu2Ac	Cu 6.3 µmol L^−1^ + Ac 60.9 mmol L^−1^
	22	1Cu3Ac	Cu 1.9 µmol L^−1^ + Ac 121.9 mmol L^−1^
	23	2Cu3Ac	Cu 4.4 µmol L^−1^ + Ac 121.9 mmol L^−1^
	24	3Cu3Ac	Cu 6.3 µmol L^−1^ + Ac 121.9 mmol L^−1^
	25	1Zn2Ac	(Zn 1.0 + Ac 60.9) mmol L^−1^
	26	1Zn3Ac	(Zn 1.0 + Ac 121.9) mmol L^−1^

Three different concentrations of Zn, provided as ZnSO_4_·7H_2_O (1.04, 2.4 and 3.5 mmol L^−1^) and Cu, provided as CuSO_4_ (1.9, 4.4, 6.3 µmol L^−1^) were used individually or in combination (experiments #1–6 and #16–18). Two concentrations of acetate (60.9 and 121.9 mmol L^−1^) were tested in addition to the recommended concentration of 12.2 mmol L^−1^ (experiments #7–9). Propionate was tested at concentrations of 10.4, 52.0 and 104.1 mmol L^−1^ without or with acetate was also tested (#10–15). In experiments #19–26 a combination of Zn or Cu with acetate was tested

*Ac* acetate, *Pr* propionate

^a^Medium in 1–9 contained an acetate concentration of 12.2 mmol L^−1^ as a standard nutrient for *M. maripaludis* growth

### Test with Cu or Zn

Three different concentrations of Zn, provided as ZnSO_4_·7H_2_O (1.04, 2.4, and 3.5 mmol L^−1^) and Cu, provided as CuSO_4_ (1.9, 4.4, 6.3 µmol L^−1^) were used for investigating the physiology and CH_4_ productivity of *M. maripaludis* (Table 2, 1–6). Therefore, two liquid stock solutions were prepared: (I) 0.17 mol L^−1^ ZnSO_4_·7H_2_O M, (II) 63 mmol L^−1^ CuSO_4_. After dispensing the m141 medium in each bottle, appropriate volumes of Zn or Cu were added to the replicates and to the negative control.

### Test with acetate and propionate

Two concentrations of sodium acetate (60.9 and 121.9 mmol L^−1^) were tested in addition to the recommended concentration of 12.2 mmol L^−1^ [74, 75] in methanogenium medium 141 (DSMZ GmbH, Germany) (Table 2, 7–9). In addition, closed batch experiments without acetate were performed. Moreover, the performance of *M. maripaludis* was tested with sodium propionate at 10.4, 52.0, and 104.1 mmol L^−1^ without or with acetate, respectively. Two stock solutions, 2.8 mol L^−1^ for acetate and a 2.6 mol L^−1^ for propionate, were prepared to add appropriate concentrations of those VFAs to the medium just before the inoculation. The positive control of experiments 10–12 contained 12.2 mmol L^−1^ of acetate and 10.4 mmol L^−1^ of propionate, while in experiments 13–15 included 12.2 mmol L^−1^ of acetate. Except for negative control, the other serum bottles were inoculated with 0.5 mL of fresh culture and incubated as specified above.

### Test with Cu and Zn

Based on the results from Cu and Zn experiments, additional tests were carried out by combining Zn (1.0 mmol L^−1^) with three distinct concentrations of Cu (0.019, 0.044, 0.063 mmol L^−1^ (Table 2, 16–18). The positive control only contained 12.2 mmol L^−1^ of acetate without adding Cu or Zn.

### Test with Cu or Zn in combination with acetate

Double variable experiments (Table 2, 19–26) were also performed by providing Cu (1.9, 4.4, 6.3 mmol L^−1^) or Zn (1.0 mmol L^−1^) in combination with acetate (60.9 and 121.9 mmol L^−1^). The positive control contained only 60.9 or 121.9 mmol L^−1^ of acetate, respectively, without adding Cu or Zn.

### Quantitative analysis

OD_578_, biomass concentration (× [g L^−1^]), specific growth rate (*µ* [h^−1^]), and volumetric CH_4_ production rate (MER [mmol L^−1^ h^−1^]) were calculated. Values for *x* were obtained by multiplying the OD values by 0.34 g L^−1^, which is the conversion factor obtained by the cell dry weight. *µ* was calculated as a linear parameter as follows: *µ* = (OD_t2 _− OD_t1_)/(*t*_2 _− *t*_1_), where *t* is the time of incubation in hours. MER was calculated, as follows [73]: ${\text{MER}}\, = \,{{\Delta n_{{{\text{CH}}_{ 4} }} } \mathord{\left/ {\vphantom {{\Delta n_{{{\text{CH}}_{ 4} }} } {\Delta t \, V}}} \right. \kern-0pt} {\Delta t \, V}}$, where $\Delta n_{{{\text{CH}}_{ 4} }}$ is the number of moles of CH_4_ produced per time per volume of gas, based on the pressure reduction in the head space after a period of culture incubation, *t* is the time, and *V* is the cultivation volume. Furthermore, the specific CH_4_ productivity (qCH_4_, [mmol g^−1^ h^−1^]), which defines the catalytic power of the biomass [8, 76] was considered. qCH_4_ is calculated by dividing MER and *x* as follows: qCH_4_ = MER/*x*. The above-described parameters will be shown as maximum values in order to compare the different growing conditions.

### VFAs analysis

VFAs were analysed at the end of each closed batch experiments. Concentrations of acetate and propionate were determined by HPLC analysis (Agilent 1100 Series HPLC System with G1362A refractive index detector, Agilent Technologies, USA). Separation was done with an IC Sep ICE-Coregel 87H3 column (Transgenomic, Nebraska, USA) with a mobile phase of 50 mol m^−3^ H_2_SO_4_ at a flow rate of 1.5 × 10^−7^ m^3^ s^−1^. Column oven and detector temperature were 65 °C and 55 °C, respectively. To remove particles from liquid suspension, the samples were prepared by Carrez precipitation and centrifugation to eliminate interfering compounds [77]. For calibration, mixed standards were prepared from pure substances at concentration levels of 10, 40, 100, 500, and 1000 ppm.

### Acetate transporter identification

The investigation on acetate uptake by *M. maripaludis* S2 through specific transport mechanism, started with the search for homology with MM_0903 protein sequence from *Methanosarcina mazei* [65]. BLAST searches were performed using the UniProt web page [78] and the NCBI platform [79]. The obtained sequences were analysed and the search for homologous was restricted to the methanococci related class to identify a candidate protein for an acetate transporter, resulting in the MMP0348 protein as the possible acetate carrier. Alignments were performed with Clustal Omega [80] and possible interactions were searched using the STRING protein–protein interaction database [72]. A protein structural model of the candidate transport protein (MMP0348) has been generated by the protein structure prediction server RaptorX [66–70].

## Additional files

**Additional file 1.** Additional figures.

**Additional file 2.** Additional materials.

## Abbreviations

Ac: acetate
ACS: acetyl-CoA synthase
BMP: biological methane production
CH_4_: methane
Co: cobalt
CO_2_: carbon dioxide
CODH: carbon monoxide dehydrogenase
Cu: copper
H_2_: molecular hydrogen
*M. maripaludis*: *Methanococcus maripaludis*
MER_max_: maximum methane evolution rate (mmol L^−1^ h^−1^)
N_2_: molecular nitrogen
OD: optical density
Pr: propionate
qCH_4_: specific methane production rate (mmol g^−1^ h^−1^)
qCH_4,max_: maximum specific methane production rate (mmol g^−1^ h^−1^)
*X*: biomass (g)
*x*: biomass concentration (g L^−1^)
Zn: zinc
*µ*: specific growth rate (h^−1^)
*µ*_max_: maximum specific growth rate (h^−1^)
