# Supplementary material for: The physiological effect of heavy metals and volatile fatty acids on Methanococcus maripaludis S2
Source: Biotechnol Biofuels. 2018 Nov 2;11:301. doi: 10.1186/s13068-018-1302-x (PMC6214177; doi:10.1186/s13068-018-1302-x)

**Figure S1.** **Adaptation of *M. maripaludis* culture to 141 media without Zn and Cu (m141(Zn,Cu)), without vitamins, Zn, and Cu (m141)**. OD_578 nm_ **(A**, **B)** and MER (**C, D**) of *M. maripaludis* (I and II generation) in *modified* and standard 141 medium DMSZ.


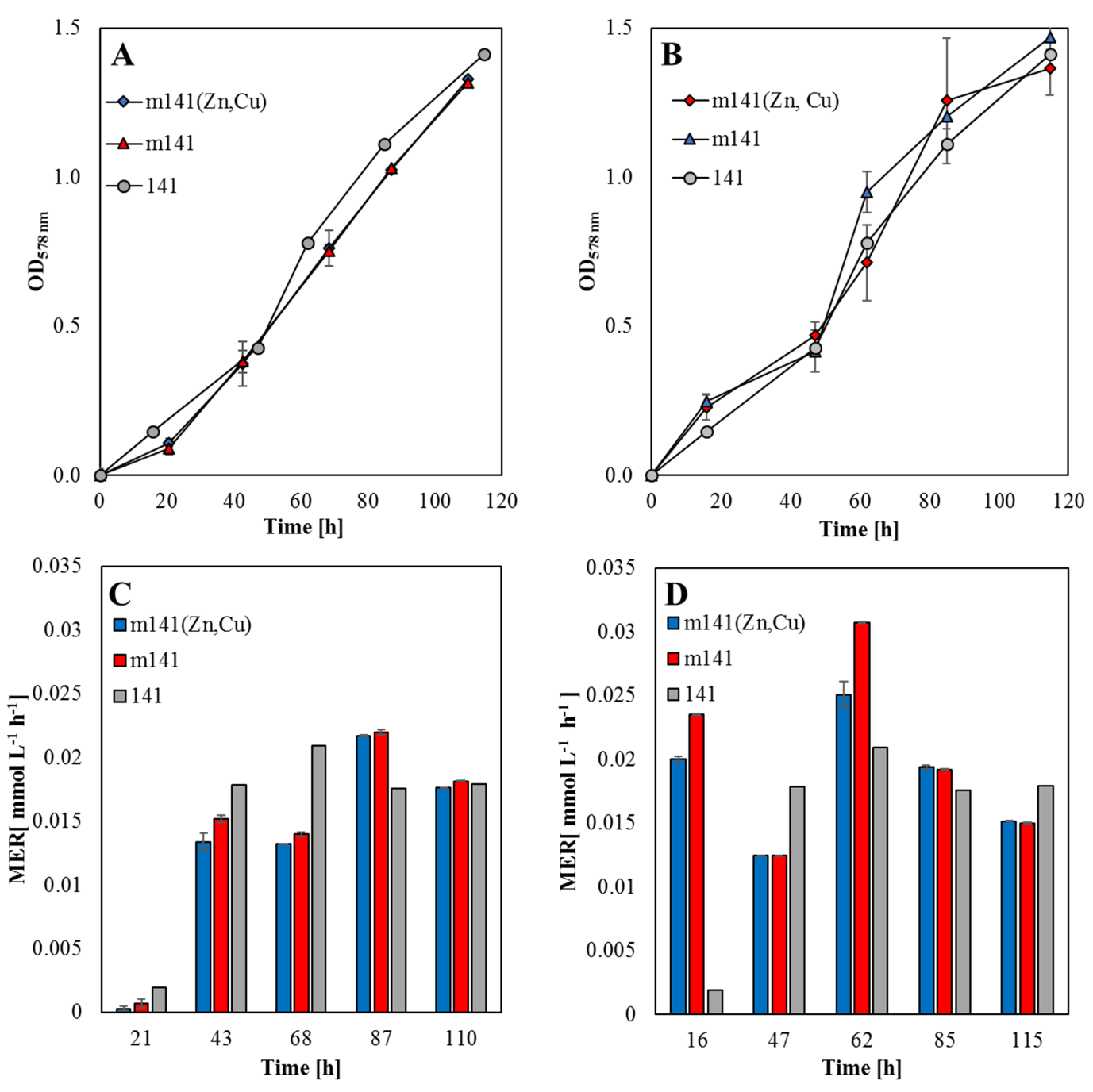


**Figure S2.** OD_578 nm_ curves and MER histograms of *M. maripaludis* at 37°C, 140 rpm, 2.9 bar. *M. maripaludis* was grown on acetate as the sole carbon source (acetate 12.2, 60.9, 121.9) mmolL^-1^and on acetate plus carbonate (acetate 12.2 +, 60.9 + , 121.9 +) mmolL^-1^ as positive control.


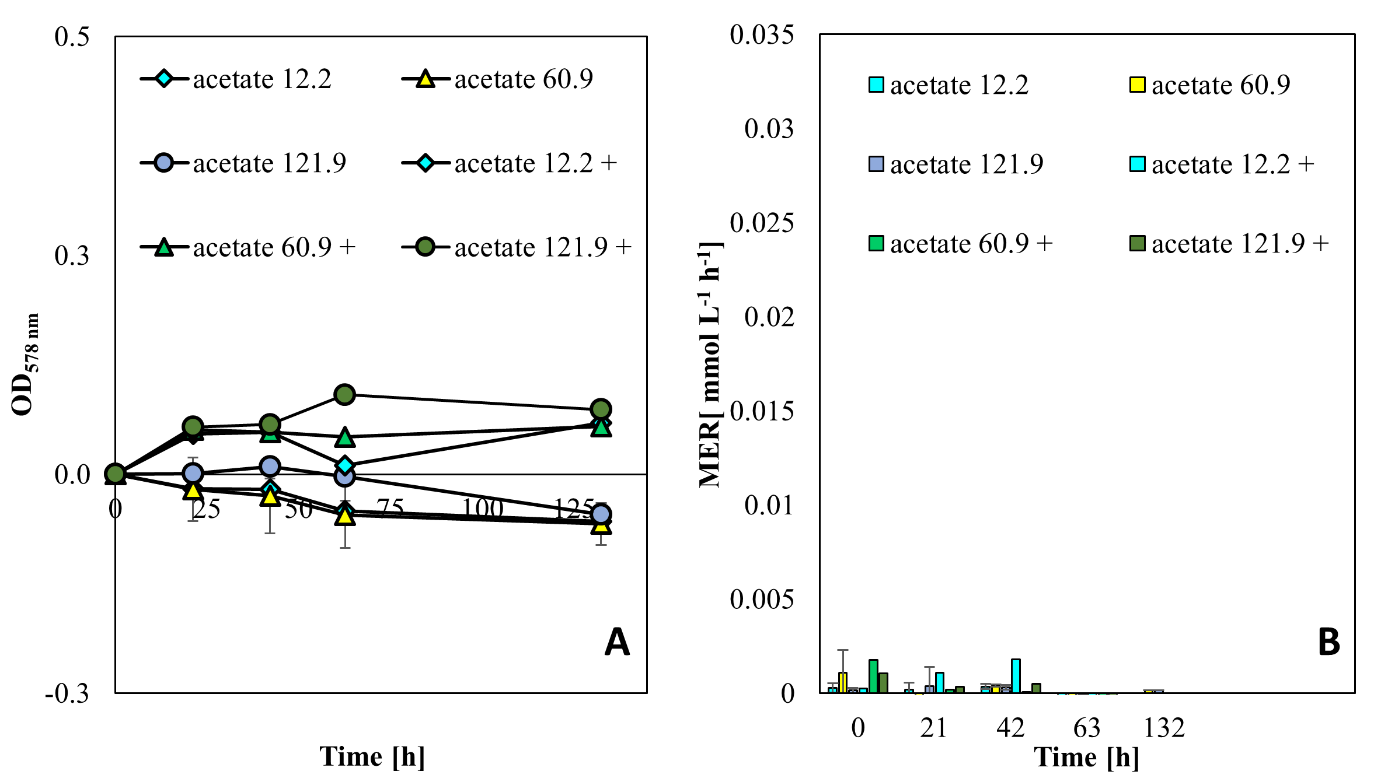

Supplement: Supplementary file 1 — Additional file 1. Additional figures. [file 13068_2018_1302_MOESM1_ESM.docx]
