# Supplementary material for: The physiological effect of heavy metals and volatile fatty acids on Methanococcus maripaludis S2
Source: Biotechnol Biofuels. 2018 Nov 2;11:301. doi: 10.1186/s13068-018-1302-x (PMC6214177; doi:10.1186/s13068-018-1302-x)
Supplement: Supplementary file 2 — Additional file 2. Additional materials. [file 13068_2018_1302_MOESM2_ESM.docx]

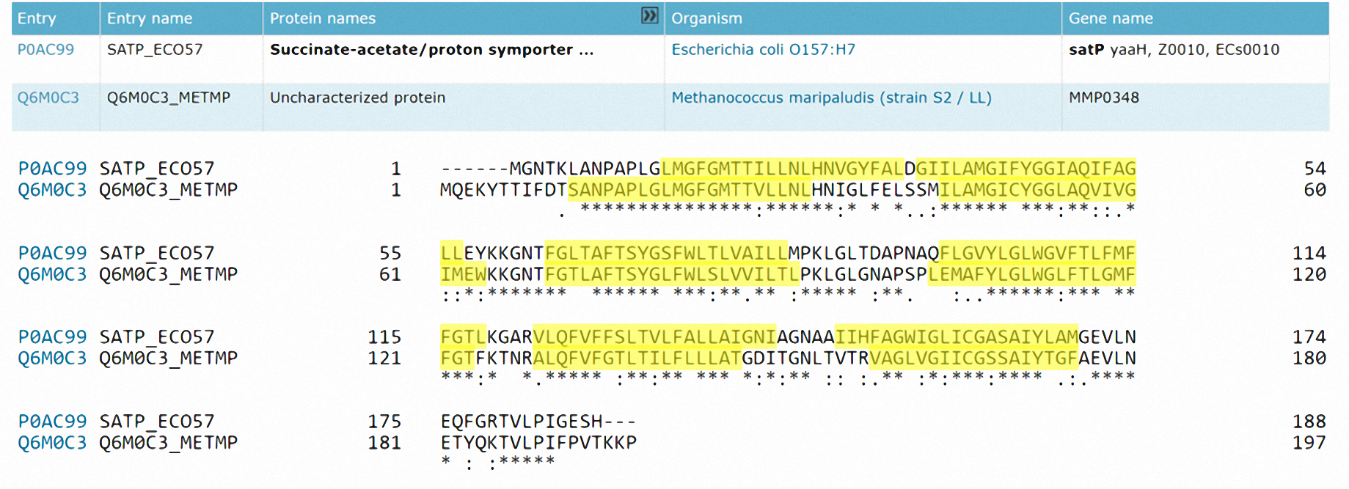
**Figure S3.** Alignment of amino acids sequences of succinate-acetate proton symporter SatP of *E.coli* O157:H7, unknown protein of *M. maripaludis* S2 candidate as Ac transporter. Yellow highlights the 6 conserved transmembrane regions of both sequences.

**Table S1.** The quality of the 3D structure model for SatP and MMP0348 was evaluated by the output values in the following tables [1-5]. P-value evaluates the relative quality of a model. The smaller the P-value, the higher quality the model. Score is the alignment score between 0 and the (domain) sequence length, with 0 indicating the worst. uGDT(GDT) measures the absolute model quality. For a protein with >100 residues, uGDT>50 is a good indicator. For a protein with <100 residues, GDT>50 is a good indicator. If a model has good uGDT (>50) but bad GDT (<50), it indicates that only a small portion of the model may be good. uSeqID is the number of identical residues in the alignment.

SATP E.COLI | sp|P0AC99|SATP_ECO57

| Rank | P-value | Score | uGDT/GDT | uSeqID/SeqID | ModelName | Template(s) |
| --- | --- | --- | --- | --- | --- | --- |
| 1 | 8.40E-06 | 211 | 179/95 | 169/90 | 5ys3A-331002_1 | 5ys3A |
| 2 | 9.40E-04 | 133 | 83/44 | 21/11 | 6eu6A-331002_1 | 6eu6A |
| 3 | 1.60E-04 | 162 | 71/38 | 22/12 | 3rkoB-331002_1 | 3rkoB |
| 4 | 7.10E-04 | 137 | 77/41 | 21/11 | 5aezA-331002_1 | 5aezA |
| 5 | 7.60E-04 | 136 | 72/39 | 19/10 | 5aexA-331002_1 | 5aexA |

1) The model and alignment files under pdbs/ and alignments/ are named after $ModelName.pdb and $ModelName.fasta, respectively.

2) Please see http://raptorx.uchicago.edu/documentation/#goto2 for the explanation of P-value, Score, uGDT(GDT) and uSeqID(SeqID).

MMP0348 | mmp0348

| Rank | P-value | Score | uGDT/GDT | DuSeqID/SeqID | ModelName | Tamplate(s) |
| --- | --- | --- | --- | --- | --- | --- |
| 1 | 7.60E-04 | 132 | 83/42 | 23/12 | 6eu6A-318610_1 | 6eu6A |
| 2 | 6.50E-04 | 135 | 77/39 | 20/10 | 5aezA-318610_1 | 5aezA |
| 3 | 1.70E-04 | 157 | 73/37 | 19/10 | 3rkoB-318610_1 | 3rkoB |
| 4 | 7.20E-04 | 133 | 72/37 | 26/13 | 2b2fA-318610_1 | 2b2fA |
| 5 | 8.20E-04 | 131 | 74/37 | 21/11 | 5aexA-318610_1 | 5aexA |

1) The model and alignment files under pdbs/ and alignments/ are named after $ModelName.pdb and $ModelName.fasta, respectively.

2) Please see http://raptorx.uchicago.edu/documentation/#goto2 for the explanation of P-value, Score, uGDT(GDT) and uSeqID(SeqID).

**References**

1. Källberg M, Wang H, Wang S, Peng J, Wang Z, Lu H, et al. Template-based protein structure modeling using the RaptorX web server. Nat Protoc. Nature Publishing Group; 2012; 7:1511–22.

2. Ma J, Peng J, Wang S, Xu J. A conditional neural fields model for protein threading. Bioinformatics. Oxford University Press; 2012; 28:59–66.

3. Ma, J., Wang, S., Zhao, F., and Xu, J. Protein threading using context-specific alignment potential. Bioinformatics. 2013; 29:257–265.

4. Peng, J., and Xu, J. A multiple-template approach to protein threading. Proteins Struct. Funct. Bioinforma. 2011; 79:1930–1939.

5. Peng, J., and Xu, J. Raptorx: Exploiting structure information for protein alignment by statistical inference. Proteins Struct. Funct. Bioinforma. 2011; 79:161–171.
